# Seasonal and spatial variations of *Synechococcus* in abundance, pigment types, and genetic diversity in a temperate semi-enclosed bay

**DOI:** 10.3389/fmicb.2023.1322548

**Published:** 2024-01-11

**Authors:** Suheng Li, Yi Dong, Xiaoxia Sun, Yuan Zhao, Li Zhao, Wuchang Zhang, Tian Xiao

**Affiliations:** ^1^CAS Key Laboratory of Marine Ecology and Environmental Sciences, Institute of Oceanology, Chinese Academy of Sciences, Qingdao, China; ^2^Laboratory for Marine Ecology and Environmental Science, Qingdao National Laboratory for Marine Science and Technology, Qingdao, China; ^3^Center for Ocean Mega-Science, Chinese Academy of Sciences, Qingdao, China; ^4^University of Chinese Academy of Sciences, Beijing, China; ^5^Jiaozhou Bay Marine Ecosystem Research Station, Institute of Oceanology, Chinese Academy of Sciences, Qingdao, China

**Keywords:** *Synechococcus*, pigment types, cpcBA operon, genetic diversity, rpoC1 gene, co-dominate, temperate semi-enclosed bay

## Abstract

*Synechococcus* is abundant and globally widespread in various marine environments. Seasonal and spatial variations in *Synechococcus* abundance, pigment types, and genetic diversity were investigated based on flow cytometric analysis and high-throughput sequencing of *cpcBA* operon (encoding phycocyanin) and *rpoC1* gene (encoding RNA polymerase) in a temperate semi-enclosed bay. *Synechococcus* abundance exhibited seasonal variations with the highest value in summer and the lowest value in winter, which was consistent with temperature variation. Three pigment types of *Synechococcus* type 1, type 2, and type 3 were distinguished based on *cpcBA* operon, which displayed obvious variations spatially between the inner and the outer bay. Freshwater discharge and water turbidity played important roles in regulating *Synechococcus* pigment types. *Synechococcus* assemblages were phylogenetically diverse (12 different lineages) based on *rpoC1* gene and dominated by three core lineages S5.1-I, S5.1-IX, and S5.2-CB5 in different seasons. Our study demonstrated that *Synechococcus* abundance, pigment types, and genetic diversity displayed variations seasonally and spatially by different techniques, which were mainly driven by temperature, salinity, nutrients, and turbidity. The combination of more technical means provides more information for studying *Synechococcus* distribution. In this study, three pigment types of *Synechococcus* were discriminated simultaneously by dual lasers flow cytometer for the first time.

## Introduction

*Synechococcus* is abundant and globally widespread in various marine environments from coastal waters to open ocean, from equatorial to polar seas, and generally reaches its highest abundance in nutrient-rich coastal waters (Farrant et al., 2016; Paulsen et al., 2016; Doré et al., 2022). As an important component of the marine microbial food web, *Synechococcus* contributes significantly in carbon biomass and primary productivity (Olson et al., 1990; Flombaum et al., 2013).

*Synechococcus* assemblages are both phenotypically and phylogenetically diverse. Three main pigment types of *Synechococcus* were divided depending on different phycobiliprotein (PBP) compositions, including type 1, type 2 and type 3. Type 1 merely contains phycocyanin (PC, encoded by *cpcBA* operon), which binds the phycocyanobilin (PCB, *A*_max_ = 620 nm). Type 2 contains both PC and phycoerythrin-I (PE-I, encoded by *cpeBA* operon), which binds both PCB and phycoerythrobilin (PEB, *A*_max_ = 550 nm). In addition to PC and PE-I, type 3 also contains phycoerythrin-II (PE-II, encoded by *mpeBA*), which binds PCB, PEB and phycourobilin (PUB, *A*_max_ = 495 nm). Studies have revealed that different pigment types prefer different light niches (Voros et al., 1998; Stomp et al., 2004, 2007). Type 1 was usually abundant in turbid estuarine waters where red light dominates (Stomp et al., 2007; Wang et al., 2011). Type 2 usually appears in the coastal and shelf waters where yellow-green light prevails, whereas type 3 appears in the oceanic waters with high transparency (Wood et al., 1998). The phylogeny of *cpcBA* and *cpeBA* operons encoding for PC and PE-I, respectively, has been widely applied to distinguish different pigment types (Larsson et al., 2014; Xia et al., 2017a; Wang et al., 2021, 2022a). Based on flow cytometric analysis, PC-only (type 1) and PE-rich (type 2 + type 3) *Synechococcus* could also be discriminated with 488 nm and 640 nm lasers (Liu et al., 2014).

*Synechococcus* strains also display a wide genetic diversity (Grébert et al., 2018). Various gene markers such as 16S rRNA gene (Dufresne et al., 2008), the 16S-23S rRNA gene internal transcribed spacer (ITS, Choi and Noh, 2009), the nitrate reductase gene (*narB*, Paerl et al., 2008), the nitrogen regulatory gene (*ntcA*, Post et al., 2011), and the ribulose-1,5-bisphosphate carboxylase/oxygenase large subunit gene (*rbcL*, Chen et al., 2006), and the RNA polymerase gene (*rpoC1*, Xia et al., 2015) have been gradually developed to study the genetic diversity of *Synechococcus*. The *rpoC1* gene is able to distinguish most of the *Synechococcus* lineages, which has been proven to be a robust gene marker (Wang et al., 2022b). Based on 16S rRNA phylogeny, *Synechococcus* strains can be classified into three subclusters, labeled subclusters 5.1, 5.2, and 5.3 (namely S5.1, S5.2 and S5.3). S5.1 is the most widespread and abundant, and contains at least 20 lineages with Clades I, II, III, and IV being the most common lineages. Clades I always coexists with Clades IV in cold and temperate nutrient-rich coastal environments (Zwirglmaier et al., 2008; Tai and Palenik, 2009; Sohm et al., 2016). Clades II and III preferentially thrive in subtropical/tropical warm waters (Post et al., 2011). S5.2 is mainly found in estuarine and brackish waters, which always copes with variations in salinity (Xia et al., 2017a, 2023). It is more abundant in river-influenced coastal waters such as in the Chesapeake Bay (Chen et al., 2004, 2006; Cai et al., 2010), Pearl River estuary (Xia et al., 2015, 2017a), and Baltic Sea (Haverkamp et al., 2008). S5.3 was less studied than S5.1 and S5.2, which has been reported in various marine and freshwater environments, with relatively low abundance in the global ocean (Farrant et al., 2016; Xia et al., 2023). Environmental factors such as temperature (Doré et al., 2022), salinity (Xia et al., 2017a, 2023), and nutrients (Sohm et al., 2016) are known to influence the distribution of *Synechococcus* lineages.

Temporal and spatial variations of *Synechococcus* pigment types and phylogenetic clades have been reported in coastal and estuarine waters (Tai and Palenik, 2009; Post et al., 2011; Liu et al., 2014; Chung et al., 2015; Xia et al., 2015). The dominance of *Synechococcus* pigment types shifted from PC-rich (type 1) to PE-rich (type 2 + type 3) along salinity/turbidity gradients. In the subtropical Pearl River estuary, PC-rich *Synechococcus* dominated in a turbid estuary in summer, whereas PE-rich *Synechococcus* dominated in coastal waters all over the year (Liu et al., 2014). A similar phenomenon has also been observed in the subtropical estuary in the Gulf of Mexico (Murrel and Lores, 2004). *Synechococcus* assemblages exhibited distinct seasonal variations in the estuarine and coastal waters. A study conducted in the Pearl River estuary has shown that S5.1-II and S5.1-IX dominated in winter. Whereas in summer S5.1-II and S5.1-VI co-occurred in the coastal water and S5.2, freshwater *Synechococcus*, and *Cyanobium* co-occurred in the estuary owing to high temperature, freshwater input during summer monsoon (Xia et al., 2015). In the California Current, S5.1-II and S5.1-III co-occurred in the months leading to the *Synechococcus* spring bloom whereas S5.1-I and S5.1-IV co-dominated during the bloom (Tai and Palenik, 2009).

The temperate Jiaozhou Bay (35°8′–36°18′N, 120°04′–120°23′E) is a typical shallow semi-enclosed bay in the western Yellow Sea, southeast of Shandong Peninsula (Xing et al., 2017). The bay is connected with the Yellow Sea through a narrow bay mouth (~2.5 km). More than 10 small rivers enter the bay and become the major sources of external nutrient input. Freshwater discharge exhibits seasonal fluctuation with the highest discharge in summer and the lowest discharge in spring and winter (Cui et al., 2021). Connected with the Yellow Sea and surrounded by Qingdao City (population > 10 million), Jiaozhou Bay is affected by both natural and anthropogenic factors, such as East Asian monsoon, Yellow Sea Water Mass, seasonal freshwater inflow, half-day tidal exchange with the open sea (Feng et al., 2018) and rapid economic development, aquaculture, and pollutions from the land. Due to the interaction between natural changes and human activities, as well as long-term monitoring and systematic investigation, Jiaozhou Bay has become a “model” bay of temperate coastal ecosystem for ecological investigation (Zhao et al., 2020; Cui et al., 2021).

The importance of *Synechococcus* in the temperate Jiaozhou Bay has been realized and seasonal variations of PE-rich *Synechococcus* abundance have been studied (Zhao et al., 2005; Yang et al., 2012). However, little is known about PC-only *Synechococcus*, which has long been overlooked and might be an important component in the studied area. It is worth noting that the distribution of PC-only *Synechococcus* abundance in the temperate Jiaozhou Bay is still unknown. Studies have revealed that freshwater discharge plays an important role in determining the proportion of PC/PE *Synechococcus* in the surface water of ECS (Chung et al., 2015) and Pearl River Estuary (Xia et al., 2015). Similarly, Jiaozhou Bay is connected with several small rivers, seasonal freshwater discharge might be an important factor influencing the variation of PE-rich and PC-only *Synechococcus* in this area. It is necessary to further study *Synechococcus* in multiple aspects. Besides, the pigmentation and genetic diversity of *Synechococcus* are still unclear in this temperate coastal ecosystem. Therefore, seasonal and spatial variations of *Synechococcus* abundance, pigment types and genetic diversity were conducted among three selected stations over a seasonal cycle using dual lasers of flow cytometry and high-throughput sequencing of *cpcBA* operon (encoding phycocyanin) and *rpoC1* gene (encoding RNA polymerase). The relationship between environmental variables (temperature, salinity, turbidity, and nutrients) and *Synechococcus* abundance, pigmentation and lineages will be discussed in temperate coastal ecosystems.

## Materials and methods

### Sample collection

**Figure 1:**
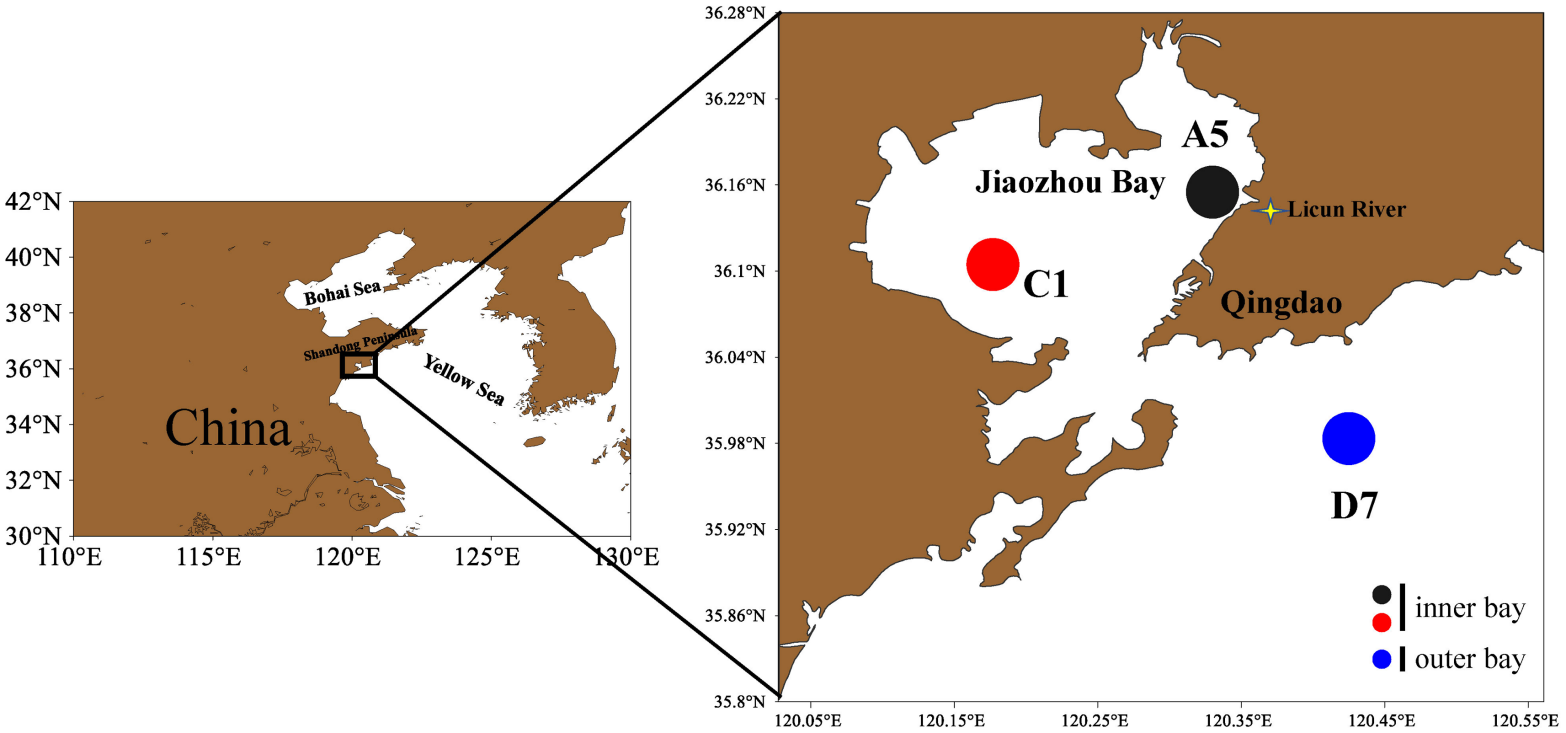
Sampling stations in Jiaozhou Bay. Stations A5 and C1 are inner bay and station D7 is outer bay.

Three stations were sampled in February (winter), May (spring), August (summer) and October (autumn) in 2021 in Jiaozhou Bay. The estuarine station A5 and central bay station C1 locate inside the bay, whereas the coastal station D7 locates outside the bay (Figure 1). All samples were taken during daytime. Surface seawater was collected at each station and divided for analysis. The samples for flow cytometric analyses (4 mL) were fixed with paraformaldehyde (final concentration 1%) after collection and then frozen in liquid nitrogen until analysis in the laboratory (Marie et al., 2000). For molecular study, 1.2 L of water was pre-filtered by 200 μm sieve, then filtered onto a 0.22 μm (50 mm) mixed cellulose membrane. The membrane was flash-frozen in liquid nitrogen and then frozen at −80°C until used for DNA extraction.

Temperature and salinity were measured by a AAQ1183-1F CTD probe (Alec, Japan). Chlorophyll *a* (Chl *a*) concentration was determined using a Turner Designs model-10 fluorometer. The measurement of nutrient concentrations (NO_3_^−^, NO_2_^−^, NH_4_^+^ and PO_4_^3−^) were conducted by a QuAAtro-SFA Analyzer (Bran-Lubbe Co., Germany). The concentration of suspended particulate matter (SPM) was measured using the method outlined by Shi et al. (2023). SPM data in October was missing (unsampled). All of the temperature, salinity, Chl *a*, nutrients, and SPM concentrations data were provided by the Jiaozhou Bay National Marine Ecosystem Research Station.

### Flow cytometric analysis of *Synechococcus* abundance

**Figure 2:**
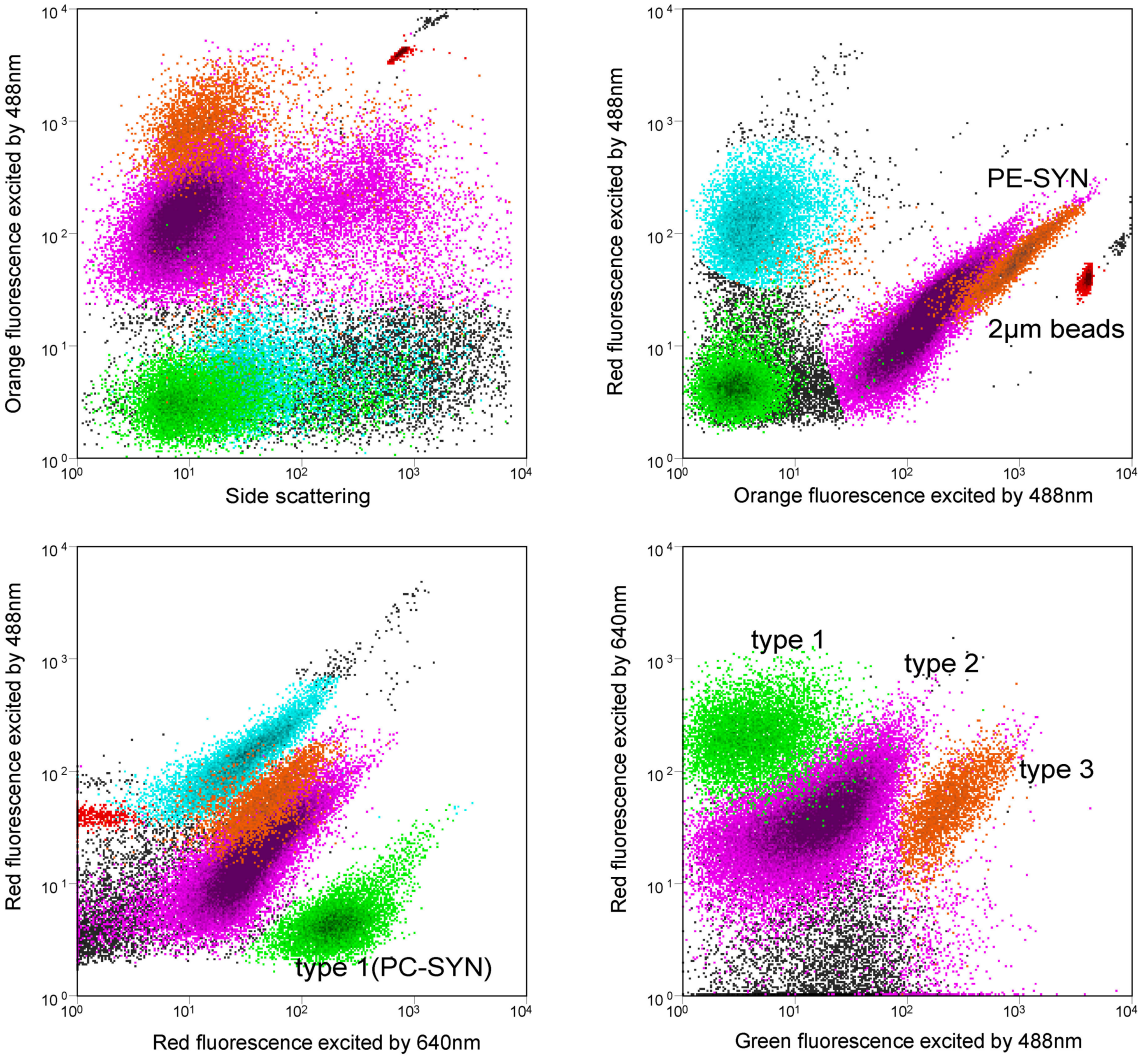
Flow cytometric signatures of type 1, type 2, and type 3 *Synechococcus* using a flow cytometer with 488 nm and 640 nm dual lasers. Orange fluorescence represents phycoerythrin (PE); Red fluorescence excited by 488 nm laser indicates chlorophyll *a*, and red fluorescence excited by 640 nm comes from phycocyanin (PC). Note that PUB-containing cells (type 3) have relatively higher green fluorescence than the non-PUB-containing cells (type 2).

*Synechococcus* cells were detected using a BD FACSJazz^™^ flow cytometer (Becton Dickinson) equipped with dual lasers of 488 nm and 640 nm. Forward scatter (FSC), side scatter (SSC), 3 fluorescence signals (green: 530/40 nm, orange: 585/29 nm, red: 692/40 nm) induced by 488 nm laser and red fluorescence (660/20 nm) induced by 640 nm laser were recorded with BD FACS^™^ Sortware Sorter software. PC-only (type 1) and PE-rich (type 2 + type 3) *Synechococcus* were distinguished from other eukaryotic picoplankton by orange fluorescence and red fluorescence induced by 488 nm laser. No *Prochlorococcus* was detected in Jiaozhou Bay. Type 1 (PC-only) *Synechococcus* was recognized by red fluorescence induced by 488 nm and 640 nm, respectively (Liu et al., 2014). Type 1, type 2, and type 3 *Synechococcus* were separated at the same time using green fluorescence induced by 488 nm and red fluorescence induced by 640 nm (Figure 2). Fluorescent beads (2 μm, Polysciences) were added to each sample as the internal standard. Flow cytometric data were analyzed with Flowjo V10 software.

### DNA extraction, PCR amplification

DNA was extracted according to the phenol-chloroform-isoamyl alcohol (25:24:1) method in Haverkamp et al. (2008). For the *cpcBA* operon, the PCR process followed the protocol of Xia et al. (2017a). The *cpcBA* operon was amplified by PCR (94°C for 5 min, followed by 40 cycles at 94°C for 30 s, 55°C for 30 s, and 72°C for 60 s and a final extension at 72°C for 1 min) using primers SyncpcB-Fw (5’-ATGGCTGCTTGCCTGCG-3′) and SyncpcA-Rev (5’-ATCTGGGTGGTGTAGGG-3′).

For the *rpoC1* gene, a nested PCR process was used (Muhling et al., 2006). Two independent PCRs used the same procedure (95°C for 5 min, followed by 30 cycles at 95°C for 60 s, 51°C for 60 s, and 72°C for 60 s and a final extension at 72°C for 10 min) with different primer sets. The first round’s primers were *rpoC1*-N5 and the C-terminal, while the second round’s primers were *rpoC1*-39F (5’-GGNATYGTYTGYG AGCGYTG) and *rpoC1*-462R (5’-CGY AGRCFCTTGRTCAGCTT) (Muhling et al., 2006; Xia et al., 2015), where barcode is an eight-base sequence unique to each sample.

Amplicons were extracted from 2% agarose gels and purified using the AxyPrep DNA Gel Extraction Kit (Axygen Biosciences, Union City, CA, United States) according to the manufacturer’s instructions.

### Library construction and sequencing

SMRTbell libraries were prepared from the amplified DNA by blunt-ligation according to the manufacturer’s instructions (Pacific Biosciences). Purified SMRTbell libraries from the Zymo and HMP mock communities were sequenced on dedicated PacBio Sequel II 8 M cells using the Sequencing Kit 2.0 chemistry. All amplicon sequencing was performed by Shanghai Biozeron Biotechnology Co. Ltd. (Shanghai, China).

### Processing of sequencing data

PacBio raw reads were processed using the SMRT Link Analysis software version 9.0 to obtain demultiplexed circular consensus sequence (CCS) reads with the following settings: minimum number of passes = 3, minimum predicted accuracy = 0.99. Raw reads were processed through SMRT Portal to filter sequences for length (<300 bp and >700 bp) and quality. Sequences were further filtered by removing barcode, primer sequences, chimeras and sequences if they contained 10 consecutive identical bases. OTUs were clustered with 95% (*cpcBA*); 97% (*rpoC1*), similarity cutoff using UPARSE (version 7.1 http://drive5.com/uparse/) and chimeric sequences were identified and removed using UCHIME (Amato et al., 2013). Representative sequences of OTU were identified using BLASTn against the nt database with an expectation value 1e-50, of which not belonging to Cyanobacteria were picked out. Reference sequences were listed in the Supplementary Tables S1, S2. There are three copies of the *cpcBA* operon in the genomic sequence of type 1 *Synechococcus* (Six et al., 2007; Xia et al., 2017a). Regarding calculation of the relative abundance of each *Synechococcus* pigment type, the OTU numbers of type 1 were divided by three. For *rpoC1* gene, sequences with less than 90% consistency with the reference sequence are assigned as unclassified.

### Phylogenetic analysis of the *rpoC1* and *cpcBA* sequences

The representative sequences of the 30 and 50 most abundant OTUs for the *cpcBA* operon (covered 99.8% of total reads) and *rpoC1* gene (covered 97.9% of total reads) were aligned with the reference sequence (Supplementary Tables S1, S2). Maximum likelihood phylogenetic trees were constructed by Mega-X with the model T92 + G (200 bootstraps) and GTR + G + I (200 bootstraps), respectively (Xia et al., 2017a). A heatmap showing the relative abundance of each OTU was formed with TBtools (Wang et al., 2021).

### Statistical analyses

The relative abundance of each pigment type and lineage was transformed by square root transformation. Environmental data were transformed by square root transformation and then normalized. Season and station variations of pigment composition and *Synechococcus* assemblages structure were investigated by the UPGMA (unweighted pair-group method with arithmetic means) cluster analysis based on the Bray–Curtis similarity matrix. ANOSIM was used to test differences in pigment composition and *Synechococcus* assemblage structure between groups. SIMPER (similarity percentage procedure) analysis was used to ascertain *Synechococcus* pigment type contributing most to the inner-outer area *Synechococcus* pigment composition dissimilarities and *Synechococcus* lineage contributing most to the four-groups *Synechococcus* assemblage dissimilarities. All the above analyses were conducted by PRIMER 6 (Clarke, 1993). The Spearman correlation analysis of *Synechococcus* abundance and environmental variables was processed by Past 4.06b. The relationships between environmental parameters and pigment composition and *Synechococcus* assemblage were studied by Mantel test using the OmicStudio tools^1^ and redundancy analysis (RDA) using CANOCO V5, Monte Carlo permutation tests (500 permutations) can be used to test significant correlations of environmental variables with assemblage structure (Cookson et al., 2006).

### Accession numbers

All sequences obtained from this study have been deposited in the National Center for Biotechnology Information (NCBI) Sequence Read Archive (SRA). The BioProject accession number PRJNA996355 (based on *cpcBA*) and PRJNA995918 (based on *rpoC1*).

## Results

### Environmental conditions

**Figure 3:**
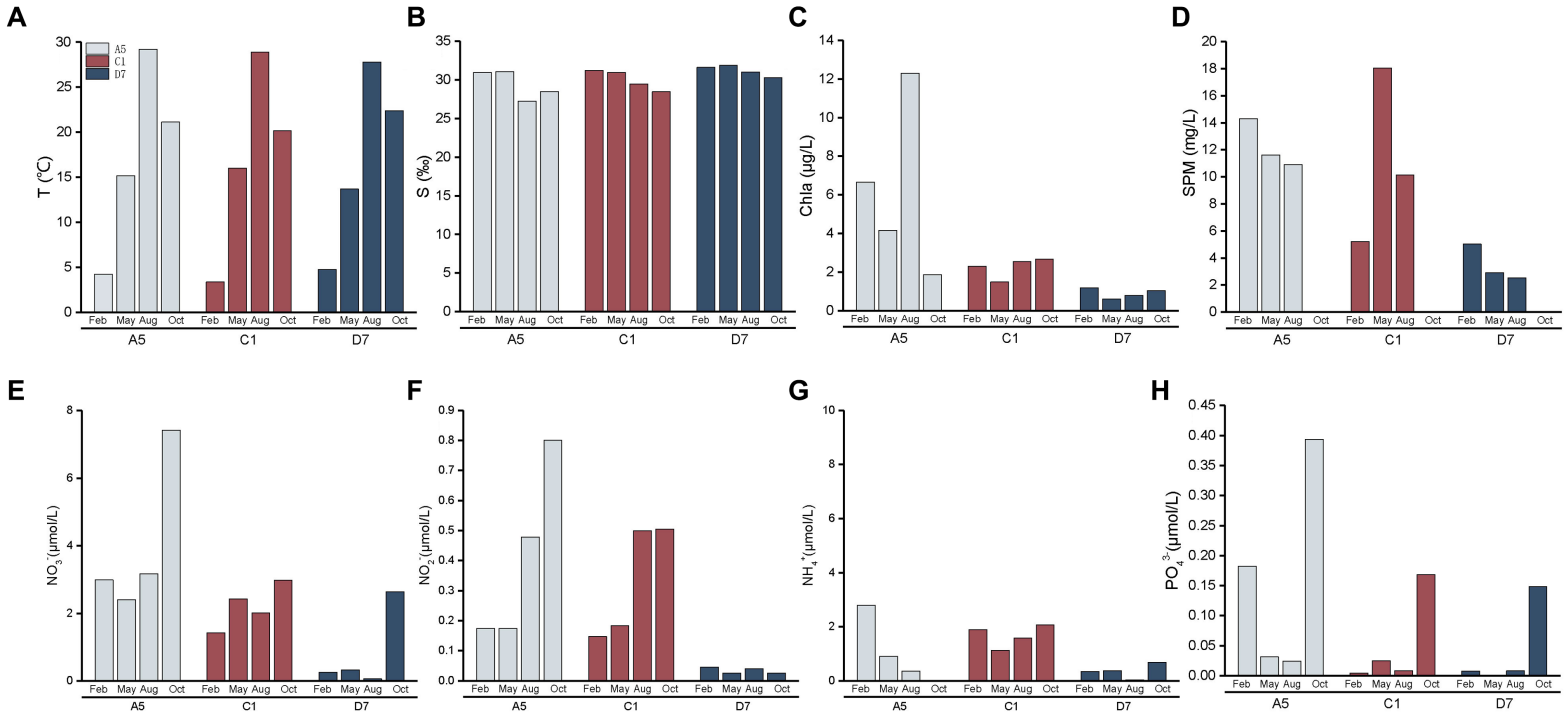
Spatial and seasonal variations of environment parameters **(A)**: temperature, **(B)**: salinity, **(C)**: Chl *a*, **(D)**: suspended particulate matter, **(E)**: NO_3_^−^, **(F)**: NO_2_^−^, **(G)**: NH_4_^+^, **(H)**: PO_4_^3−^ in Jiaozhou Bay. The data of suspended particulate matter was missing in October.

Seasonal and spatial variations of environmental variables in Jiaozhou Bay are shown in Figure 3. Three stations A5, C1, and D7 exhibited similar hydrographic features in temperature and salinity. Temperature was highest in summer (August) (27.7–29.2°C) and lowest in winter (February) (3.4–4.7°C). Salinity in summer and autumn (October) was slightly lower than in winter and spring (May). The lowest salinity (27.2) in the estuarine station A5 in summer was mainly attributed to freshwater discharge and summer rainfall. Spatial variations of Chl *a*, SPM and nutrient concentrations (NO_3_^−^, NO_2_^−^, NH_4_^+^, and PO_4_^3−^) differed among three stations with A5 > C1 > D7. SPM was much higher at stations A5 and C1, indicating higher turbidity of the stations. Seasonally, the highest nutrient concentrations including NO_3_^−^, NO_2_^−^, NH_4_^+^, and PO_4_^3−^ were mostly detected in autumn, suggesting a relatively nutrient-rich environment in autumn.

### *Synechococcus* abundance based on flow cytometry

**Figure 4:**
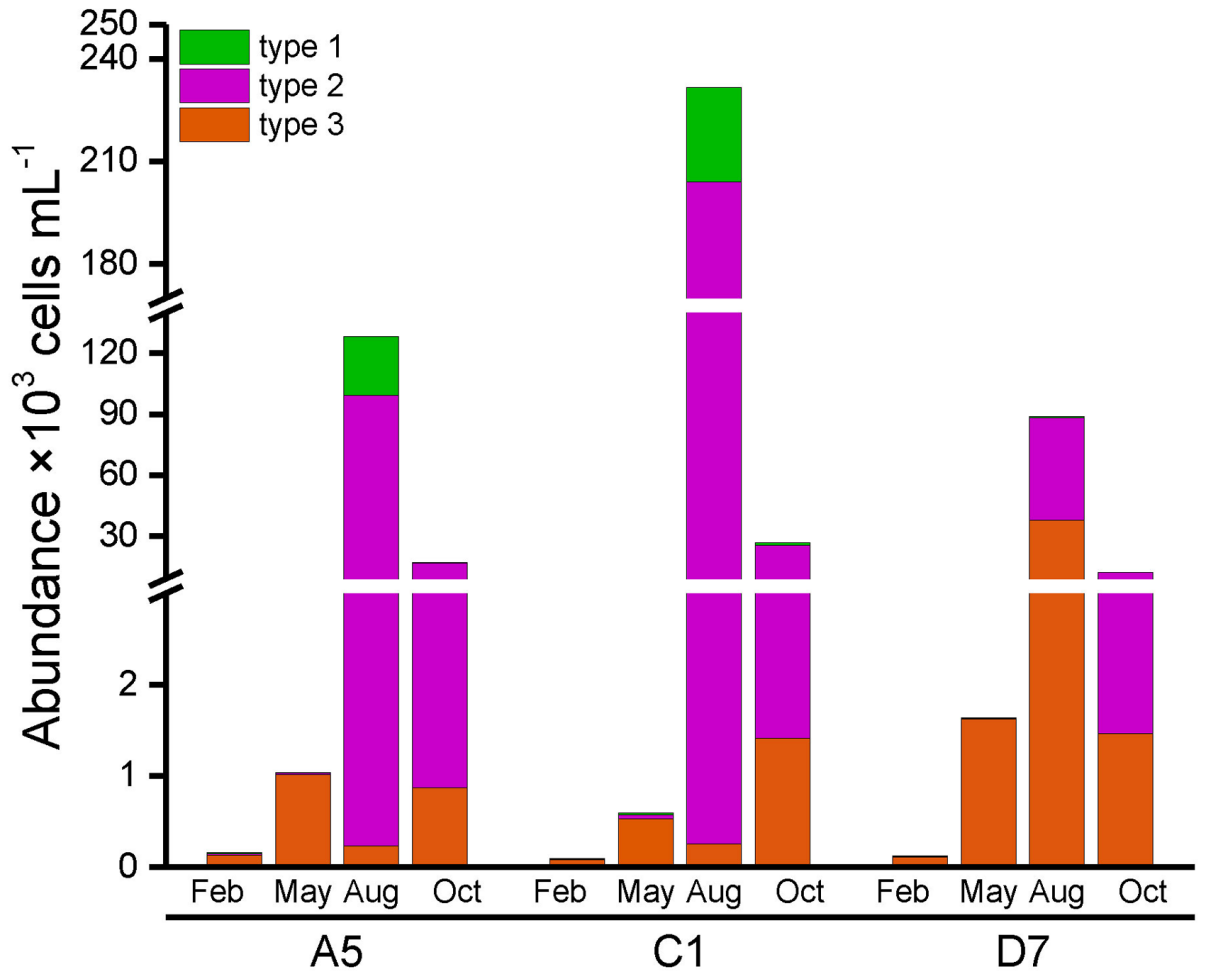
*Synechococcus* abundance measured by flow cytometric analysis.

Total abundances of *Synechococcus* measured by flow cytometry displayed similar seasonal patterns among three stations (Figure 4). Total *Synechococcus* had the highest abundances in summer and the lowest abundances in winter, which was consistent with temperature variation (Figure 3A). Our data showed that abundances in summer were 2–3 orders of magnitude higher than those in winter. Total *Synechococcus* abundances were positively correlated with temperature (*r* = 0.93, *p* < 0.01; Supplementary Table S3). The highest abundance of *Synechococcus* occurred at station C1 in summer (231.54 × 10^3^ cells mL^−1^), and the lowest abundance occurred at station C1 in winter (0.1 × 10^3^ cells mL^−1^) (Figure 4). Flow cytometry allowed us to separate three pigment types of *Synechococcus*, namely type 1 (PC-only *Synechococcus*), type 2, and type 3 (PE-containing *Synechococcus*) (Figure 2). Type 2 was predominant in Jiaozhou Bay and exhibited similar seasonal pattern as total *Synechococcus* abundance, while type 1 and type 3 had the lower abundance with different seasonal variations compared to type 2. Type 2 was more abundant in summer and autumn, with abundances ranging from 10.71 × 10^3^ cells mL^−1^ to 203.76 × 10^3^ cells mL^−1^. In winter and spring, type 2 was almost negligible (≦41 cells mL^−1^). Type 1 was less abundant than type 2 with higher abundances in summer. In winter and spring, type 1 was nearly undetected (<20 cells mL^−1^). Type 3 was less abundant than type 2 and type 1. Seasonal variation of type 3 abundance was similar to type 2 while varied among three stations. Type 2 and type 1 were more abundant at stations A5 and C1, whereas type 3 was slightly more abundant at station D7 (Figure 4). The significance test for the comparison of *Synechococcus* abundance is shown in Supplementary Table S4.

In general, type 1 was more abundant in summer at stations A5 and C1. Type 2 dominated in summer and autumn, which seemed more important at stations A5 and C1. Spearman correlation analysis showed that type 1 and type 2 were positively correlated with temperature (*r* = 0.84, *p* < 0.01 and *r* = 0.94, *p* < 0.01, respectively, Supplementary Table S3), and negatively correlated with salinity (*r* = −0.86, *p* < 0.01 and *r* = −0.80, *p* < 0.01 respectively, Supplementary Table S3). On the contrary, type 3 dominated in winter and spring, and was more important at station D7 (Figure 4).

### *Synechococcus* pigment composition based on *cpcBA* gene

The *cpcBA* gene was sequenced for 12 samples (4 seasons × 3 stations) from Jiaozhou Bay. A total of 125,309 high-quality sequences were generated with an average of 10,442 sequences per sample. The diversity of *Synechococcus cpcBA* operon was estimated by the Shannon diversity index, which ranged from 1.21 to 3.98 (Supplementary Table S5). In general, the diversity of *Synechococcus cpcBA* operon differed among stations, with A5 and C1 showing higher diversity and D7 showing lower diversity. Seasonally, the diversity of *Synechococcus cpcBA* operon at station A5 was highest in autumn (3.11) and lowest in spring (1.82). Similarly, station C1 had the highest and lowest diversities in winter (3.98) and spring (1.59), respectively. Station D7 showed lower and less obvious variations, with highest and lowest diversities in summer (2.63) and winter (1.21), respectively.

**Figure 5:**
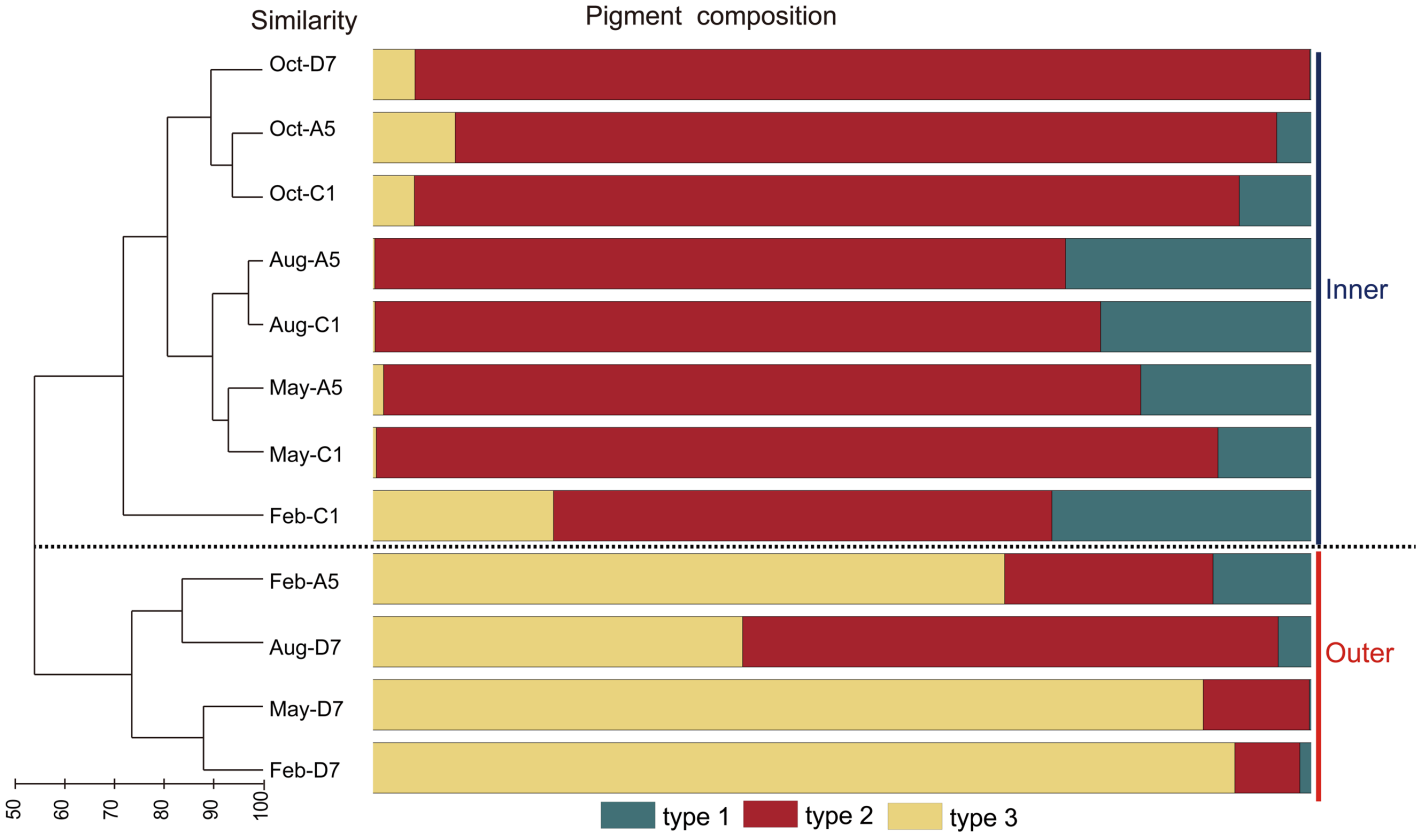
Results of UPGMA cluster analysis. *Synechococcus* pigment composition based on the relative abundance of each *Synechococcus* pigment type. The samples formed two groups, inner and outer.

In the phylogenetic tree based on the *cpcBA* operon, three well-separated clusters (type 1, type 2 and type 3) were formed (Supplementary Figure S1). According to the similarity of pigment composition recognized by UPGMA clustering, 12 samples were classified into two groups: the inner bay and the outer bay (Figure 5). The pigment composition of the two groups were significantly different (ANOSIM, *p* < 0.01). The group in the inner bay was mainly composed of samples from stations A5 and C1, except for Oct-D7. In the inner bay, type 2 was the dominant pigment type, followed by type 1 in summer and spring. The group in the outer bay was mostly composed of samples from station D7, except for Feb-A5. In the outer bay in winter and spring, type 3 was the major pigment type, and followed by type 2. Whereas in sample Aug-D7, type 2 and type 3 co-dominated (Figure 5). The dissimilarities of SIMPER analysis identified the contribution of each pigment type in the inner bay and outer bay. The average dissimilarity between two groups was 39.9%. In the inner bay (station A5 and station C1), *Synechococcus* pigment types were mainly composed of type 2 and type 1. In the outer bay (station D7), it was mainly composed of type 3 and type 2 (Table 1).

**Table 1:** *Synechococcus* pigment types that contribute most to the *Synechococcus* pigment composition dissimilarities between the inner (stationC1 and stationA5) and outer (station D7) bay stations in Jiaozhou Bay (SIMPER results with a cut-off at 100% cumulative contribution).

*Synechococcus* pigment types	Contribution (%)	Cumulative contribution (%)	Average abundance (%)	
			Inner	Outer
Type 3	54.21	54.21	19.66	73.37
Type 2	24.35	78.56	69.56	56.48
Type 1	21.44	100	31.11	9.97

Three pigment types of *Synechococcus* type 1, type 2, and type 3 were distinguished based on the phylogenetic analysis of *cpcBA* operon. On the other hand, flow cytometric analysis was also able to separate type 1, type 2, and type 3 (Figure 2; Supplementary Figure S2A) (Olson et al., 1988; Liu et al., 2014). Comparing the relative abundance of type 1, type 2, and type 3 with the two mentioned methods, 9 of 12 samples exhibited a similar distribution pattern. The dissimilarity mainly comes from three samples in the winter and spring in the inner bay (Feb-C1, May-C1, and May-A5) with low abundances (Supplementary Figure S2).

### *Synechococcus* genetic diversity based on *rpoC1* gene

The *rpoC1* gene was sequenced for 12 samples (4 seasons × 3 stations) in Jiaozhou Bay. A total of 64,031 high-quality sequences were obtained with an average of 5,336 sequences per sample. The Shannon diversity index of *Synechococcus* assemblages ranged from 3.76 to 4.87. Seasonally, the diversity of *Synechococcus* assemblages was more pronounced in autumn than in other seasons, with the highest and lowest diversity both observed in autumn. Spatially, stations C1 and A5 showed higher diversity than station D7. The Shannon diversity based on the *rpoC1* operon was much higher than that based on the *cpcBA* operon in all the samples (Supplementary Table S5).

Based on our database, 97.6% of the sequences were classified. All three marine *Synechococcus* subclusters (S5.1, S5.2, and S5.3) were detected based on *rpoC1* gene. S5.1 was most abundant in 9 samples with relative abundances ranging from 48.0 to 88.3%. S5.2 was less abundant than S5.1, whereas in samples May-C1, Aug-C1, and Aug-A5, S5.2 outnumbered S5.1 with relative abundances ranging from 50 to 61.4%. S5.3 was a minor component and merely detected in autumn with relative abundances from 6.7 to 18.1%. Freshwater *Synechococcus* (FS) and *Cyanobium* were found in the inner bay with relative abundance ≤4.5% (Supplementary Table S6).

**Figure 6:**
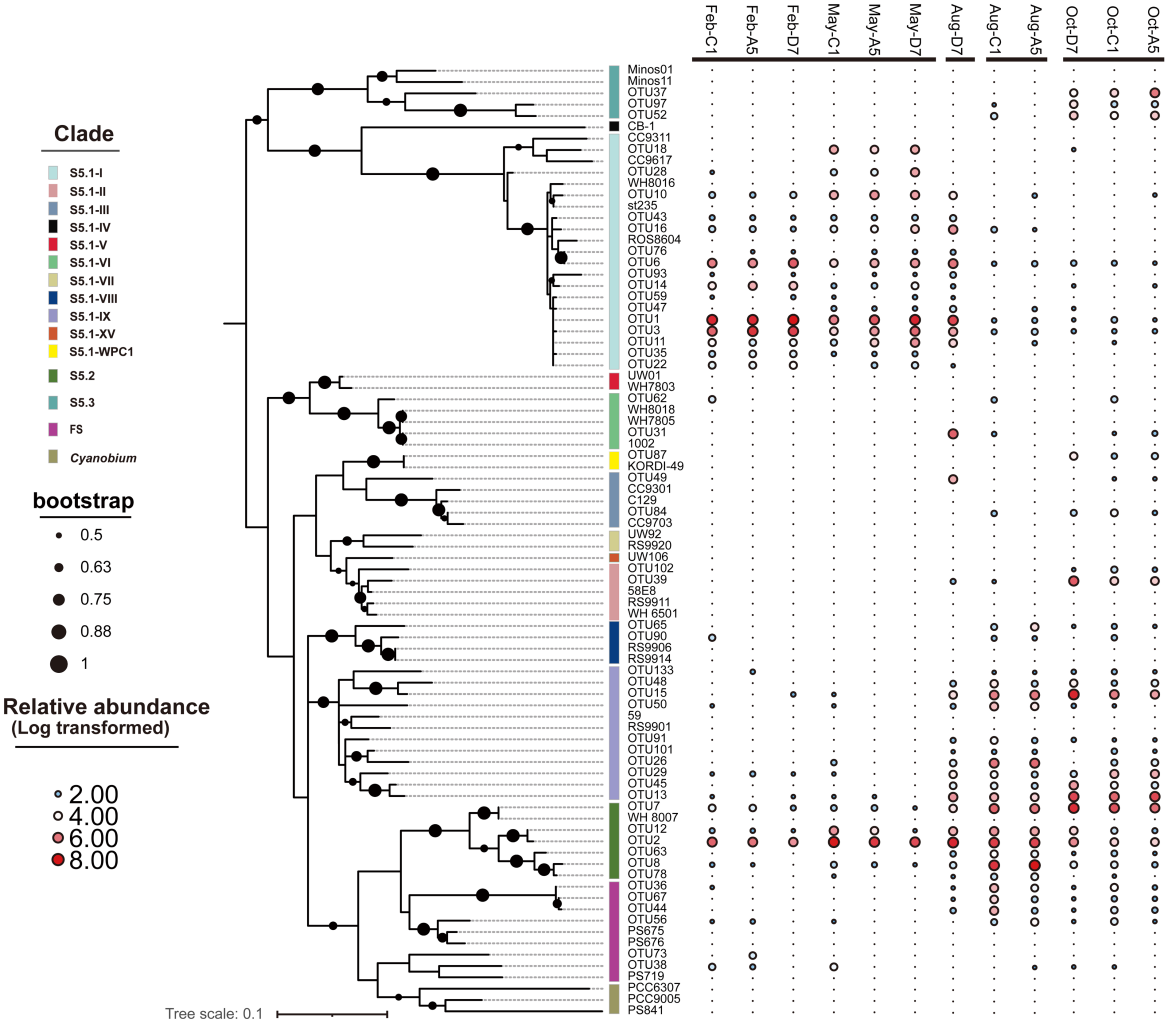
Maximum likelihood phylogenetic tree of the 50 most abundant *rpoC1* OTUs across all samples. The heatmap on the right-hand side shows the relative abundance of OTUs in each library (Log transformed). Only nodes with bootstrap values higher than 50% are shown.

In the phylogenetic tree (Figure 6), 10 clades in total were detected, with S5.1-I, S5.1-IX, and S5.2-CB5 being the most abundant lineages in Jiaozhou Bay. S5.1-I was dominant in winter and spring, whereas S5.1-IX was dominant in autumn. S5.2-CB5 was widespread and abundant (11.2–61.4%) in all the samples and predominant in summer. Most OTUs of S5.1-I in winter and spring were affiliated with *Synechococcus* sp. st235. However, OTU18 and OTU28 merely occurred in spring, which were affiliated with *Synechococcus* sp. CC9311 and CC9617. OTUs belonging to S5.1-IX in summer and autumn were affiliated with *Synechococcus* sp. RS9901 and 59. S5.1-II and S5.1-III were merely observed in summer and autumn, especially in autumn. OTUs of S5.2-CB5 were affiliated with *Synechococcus* sp. WH8007. OTU2, OTU7 and OTU12 of S5.2-CB5 occurred in all the samples, whereas OTU8 and OTU63 mainly occurred in summer and autumn. OTUs of S5.3 were affiliated with *Synechococcus* sp. Minos01 and Minos11, which merely occurred in autumn.

**Figure 7:**
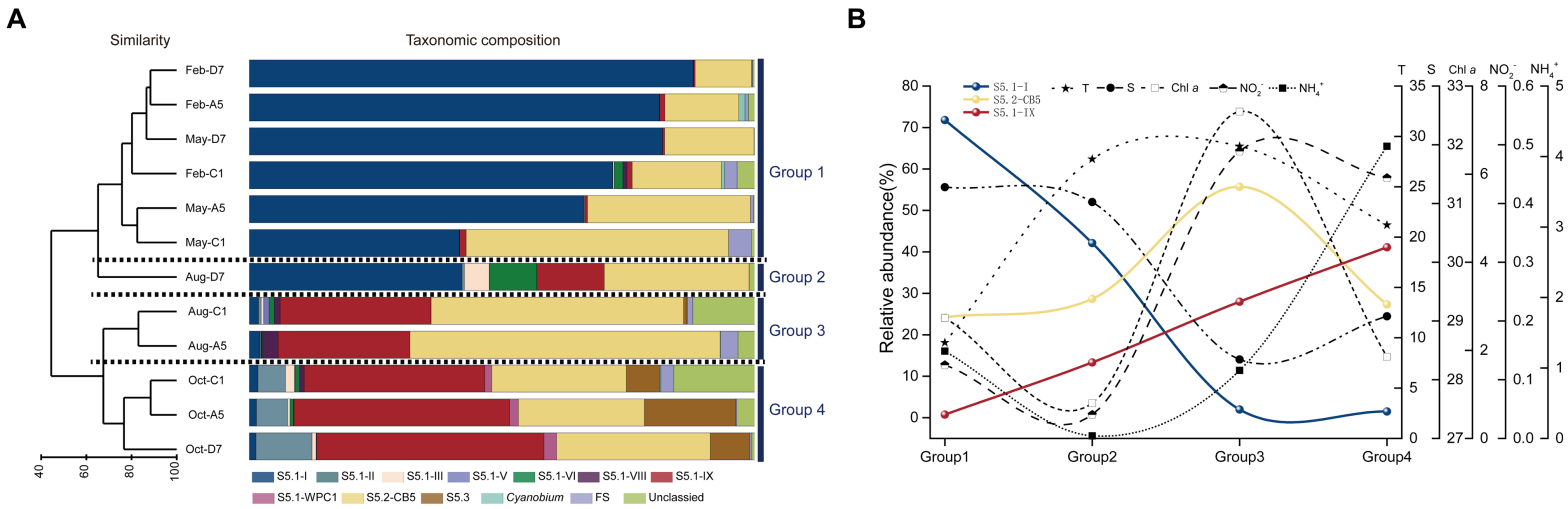
Results of UPGMA cluster analysis. *Synechococcus* assemblages composition based on the relative abundance of each *Synechococcus* lineage. The samples formed four groups, Group 1, Group 2, Group 3, and Group 4 **(A)**. The trends in the relative abundance of dominant lineages S5.1-I, S5.1-IX, and S5.2-CB and typical environmental factors-T, S, Chl *a*, NO_2_^−^, NH_4_^+^ in the above four groups. The data in each group is the average of the total sample data in the corresponding group. T, temperature; S, salinity **(B)**.

The *Synechococcus* assemblages structure was recognized by UPGMA clustering. According to the similarity of taxonomic composition, 12 samples were classified into four groups: Group 1-samples in winter and spring, Group 2-sample in summer at station D7 (Aug-D7), Group 3-samples in summer at stations C1 and A5 (Aug-C1 and Aug-A5), Group 4-samples in autumn (Oct-C1, Oct-A5, and Oct-D7) (Figure 7A). In Group 1, S5.1-I was predominant in most samples and followed by S5.2-CB5. They co-dominated in winter and spring with relative total abundances ranging from 89.6 to 99.5%. The importance of S5.1-I decreased from winter to spring whereas S5.2-CB5 exhibited an opposite trend. In Group 2, S5.1-I and S5.2-CB5 dominated at station D7 in summer with relative abundance of 42.1 and 28.7%, respectively. Besides, S5.1-IX, S5.1-VI, and S5.1-III also contributed to the high abundance with relative abundances of 13.3, 9.4, and 4.9%, respectively. In Group 3, S5.2-CB5 and S5.1-IX co-dominated in summer with their relative total abundances of 79.8 and 87.5% at stations C1 and A5, respectively. Furthermore, S5.1-VIII and S5.1-I were also detected with proportions <3.2%. In Group 4, S5.1-IX became the core lineage, and followed by S5.2-CB5 in autumn. Other *Synechococcus* lineages including S5.1-II, S5.1-III, S5.1-WPC1 and S5.3 also made minor contributions to *Synechococcus* abundance (Supplementary Table S6).

In general, three dominant lineages of *Synechococcus* exhibited distinct seasonal variations in Jiaozhou Bay. S5.1-I decreased gradually from winter to spring (Group 1), and almost disappeared in summer (Group 3) and autumn (Group 4), except for the sample Aug-D7 (Group 2). On the contrary, S5.1-IX was negligible from winter to spring (Group 1), increased gradually from summer (Group 2, 3) and became dominant in autumn (Group 4). S5.2-CB5 was widespread in all the samples and became predominant in summer at station A5 and C1 (Group 3) (Figure 7B).

### Impact of environmental variables on *Synechococcus* pigment composition (*cpcBA*) and genetic diversity (*rpoC1*)

**Figure 8:**
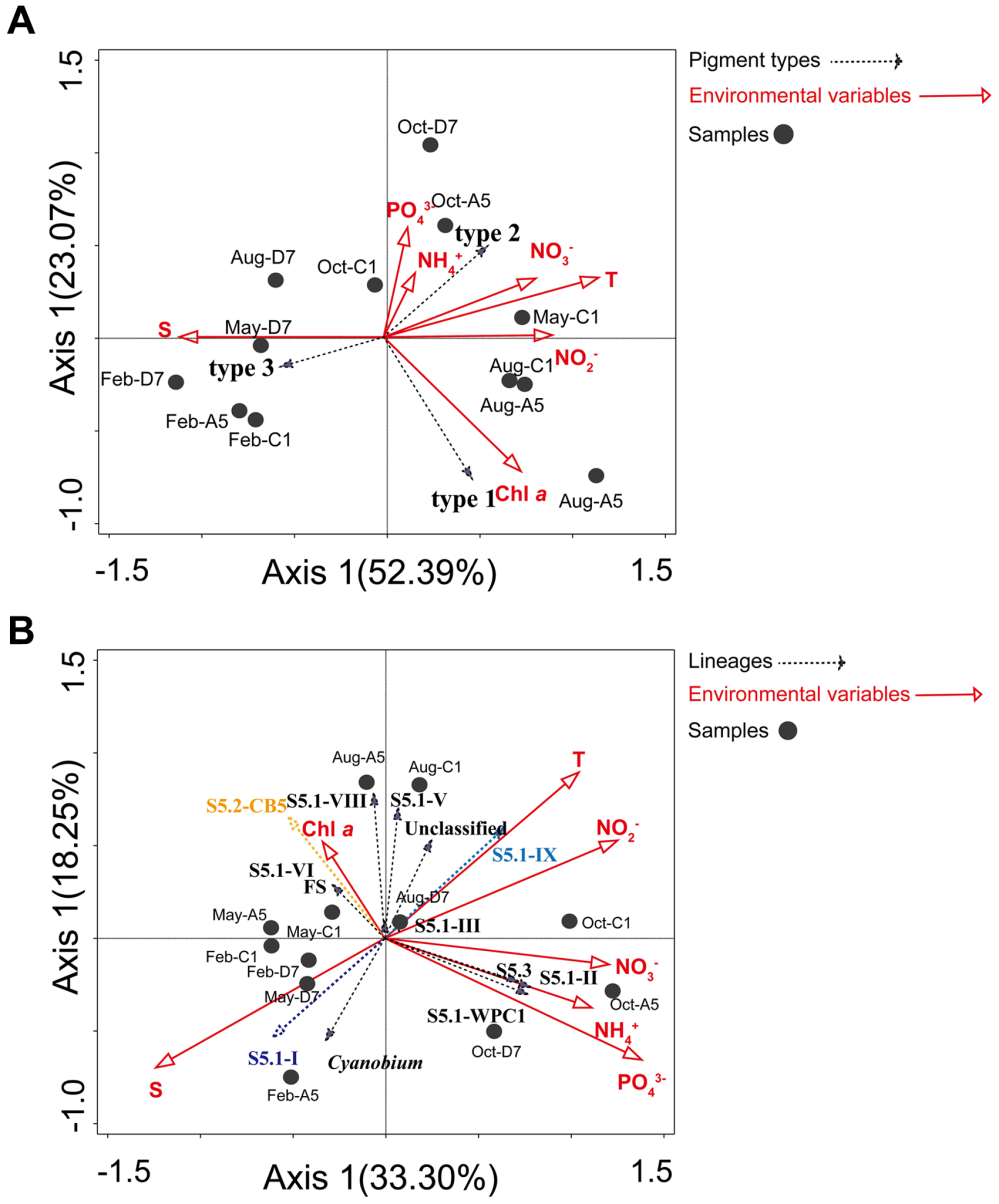
RDA analysis. **(A)** The relationship between the distribution of *Synechococcus* pigment types (*cpcBA*) and environmental factors in Jiaozhou Bay. **(B)** The relationship between the distribution of *Synechococcus* lineages (*rpoC1*) and environmental factors in Jiaozhou Bay.

Environmental variables and distribution of the *Synechococcus* pigment composition were analyzed by the Mantel test. Temperature (Mantel test, *p* = 0.014) had a significant impact on *Synechococcus* pigment composition. RDA analysis reflects the relationships between *Synechococcus* pigment types and environmental variables (Figure 8A). A Monte Carlo test (499 permutations) showed a high significance for the whole model (*p* < 0.001) and demonstrated that environmental factors affect pigment type distribution. The first two axes explained 52.4 and 23.1% of *Synechococcus* pigment variance, respectively. *Synechococcus* type 1 was positively correlated with Chl *a*. Type 2 was positively correlated with temperature, NO_3_^−^, NO_2_^−^, NH_4_^+^, and PO_4_^3−^, and negatively correlated with salinity. On the contrary, type 3 was positively correlated with salinity, and negatively correlated with temperature and NO_3_^−^, NO_2_^−^, NH_4_^+^, and PO_4_^3−^.

Environmental variables and distribution of the *Synechococcus* assemblages were analyzed by the Mantel test. Temperature (*p* < 0.05), salinity (*p* < 0.01), and PO_4_^3−^ (*p* < 0.05) had significant impacts on the distribution of *Synechococcus* assemblages. RDA analysis (Figure 8B) reflects the relationships between *Synechococcus* lineages and environmental variables. A Monte Carlo test (499 permutations) showed a high significance for the whole model (*p* < 0.001) and demonstrated that environmental factors affect lineages distribution. The first two axes explained 33.3 and 18.3% of lineages variance, respectively. S5.1-I was positively correlated with salinity, and negatively correlated with temperature and NO_2_^−^. On the contrary, S5.1-IX was positively correlated with temperature and NO_2_^−^, and negatively correlated with salinity. S5.2-CB5 and FS were positively correlated with Chl *a*, and negatively correlated with NO_3_^−^, NH_4_^+^, and PO_4_^3−^, whereas S5.1-II, S5.3, and S5.1-WPC1 were positively correlated with NO_3_^−^, NH_4_^+^, and PO_4_^3−^ and negatively correlated with Chl *a*.

## Discussion

### Distribution of *Synechococcus* abundance (FCM)

In the temperate Jiaozhou Bay, a clear seasonality in *Synechococcus* abundance was observed, with maximum abundance in summer and minimum abundance in winter (Figure 4). A strong positive correlation was found between temperature and *Synechococcus* abundance (*r* = 0.93, *p* < 0.01), indicating the importance of temperature in regulating *Synechococcus* abundance (Agawin et al., 1998; Mitbavkar et al., 2009). Similar temperature-driven pattern has been reported in Bohai Sea (Wang et al., 2022a), Chesapeake Bay (Cai et al., 2010), and Martha’s Vineyard Coastal Observatory (Hunter-Cevera et al., 2016). High temperature could support rapid growth rate of *Synechococcus* (Wang et al., 2011). In the present study, the optimum temperature range of 27.5–29.5°C in summer seemed to be favorable for high abundance of *Synechococcus* (Figure 3). Under the circumstance of global warming, surface temperature in Jiaozhou Bay showed a rising trend over the past decades. Larger phytoplankton showed a downward trend in species composition and cell abundance (Chen et al., 2023). Accordingly, phytoplankton tend to be small-sized in Jiaozhou Bay and *Synechococcus* may become more and more important in the future.

Besides temperature, salinity could be another important influencing factor. The ratio of PC-only: PE-rich *Synechococcus* abundances decreased with salinity gradient. PC-only *Synechococcus* was more abundant in lower salinity waters whereas PE-rich *Synechococcus* dominated in higher salinity waters (Wang et al., 2011; Rajaneesh and Mitbavkar, 2013; Xia et al., 2017a). Widespread coexistence of PC-only (type 1) and PE-rich (type 2 + type 3) *Synechococcus* have been found in turbid estuarine and coastal waters, such as in the Chesapeake Bay (Wang et al., 2011), Martha’s Vineyard Coastal Observatory (Hunter-Cevera et al., 2016), and Pearl River Estuary (Xia et al., 2017a). Similarly in Jiaozhou Bay, type 1 (PC-only) abundance decreased from the inner bay to the outer bay along with salinity increase mainly owing to freshwater discharge (Figures 3, 4). A negative correlation was observed between type 1 abundance and salinity, implying the importance of salinity on type 1 distribution. In the subtropical Hong Kong waters, PC-only *Synechococcus* dominated in the estuarine station in summer, whereas PE-rich *Synechococcus* dominated in the coastal waters all over the year (Liu et al., 2014). Our observations did not agree well with the results in Hong Kong waters, with a much lower abundance of type 1, perhaps owing to lower temperature and lower freshwater discharge in Jiaozhou Bay.

### Spatio-temporal variation of *Synechococcus* pigment composition (*cpcBA*)

Niche partitions of *Synechococcus* pigment types have been widely studied (Liu et al., 2014; Xia et al., 2017a; Grébert et al., 2018; Wang et al., 2022a). Type 1 is abundant in low salinity, nutrient-rich, turbid coastal and estuarine waters. Type 2 is preferred in coastal and shelf waters, whereas type 3 dominates in oligotrophic open oceans (Xia et al., 2017b; Grébert et al., 2018). The phylogeny of *cpcBA* operon (encoding PC) and *cpeBA* operon (encoding PE) were applied to distinguish *Synechococcus* pigment types. Since type 1 (PC-only *Synechococcus*) could not be recognized by *cpeBA* operon because of lacking PE, in this study *cpcBA* operon was applied and three pigment types type 1, type 2, and type 3 were thus distinguished in Jiaozhou Bay (Supplementary Figure S1). Studies have reported that different pigment types usually co-occur and one phenotype generally predominates in marine environments (Xia et al., 2017b). Similar in Jiaozhou Bay, type 1, type 2, and type 3 co-occurred, with type 2 being predominant in the majority of samples (Supplementary Figure S2B). Type 1 was more abundant in summer at stations A5 and C1 with low salinity of 27.23 and 29.47, respectively (Figure 3). Low salinity in the inner bay was mainly attributed to freshwater discharge and summer rainfall (Cui et al., 2021). Lower salinity and higher temperature seemed to be favorable for the higher growth rate of type 1 *Synechococcus* in the inner bay in summer.

Type 2 is prevalent in turbid waters, and type 3 is usually distributed in clearer water with high transparency. It has been proposed that the dominant pigment type shifted from type 2 to type 3 following a decrease in turbidity in the marine environment (Everroad and Wood, 2012; Xia et al., 2018). A similar phenomenon was observed in Jiaozhou Bay. Type 2 was predominant in the inner bay with lower salinity, higher nutrients, and higher turbidity (Figure 3; Supplementary Figure S2B). SPM in the inner bay was much higher than those in the outer bay, implying higher turbidity in the inner bay (Figure 3D). The dominance of type 2 in the inner bay indicated that it was well adapted to harvest light in the turbid waters. In the outer bay, type 3 was predominant in winter and spring with higher salinity, lower nutrients, and lower turbidity (Figure 3; Supplementary Figure S2B). The outer bay is connected to the Yellow Sea with clearer water, which is suitable for the survival of type 3. We found that the dominant pigment type not only varied with locations from the inner bay to the outer bay, but also varied with seasons from the winter to the autumn (Supplementary Figure S2B). In the outer bay, the dominant pigment type shifted from type 3 to type 2 from the winter to the autumn. In winter and spring, type 3 predominated, whereas type 2 co-dominated with type 3 in summer and even became the predominant pigment type in autumn. It is necessary to find out the possible reason. The proportion of type 3 decreased with increasing temperature (Figure 3; Supplementary Figure S2B). RDA analysis also showed that type 3 was negatively correlated with temperature (Figure 8A), implying the importance of temperature in regulating the variation of type 3. Salinity was also an important factor in the composition of *Synechococcus* pigmentation. Compared to type 1 and type 2 with a higher tolerance for salinity variations, type 3 seemed to be less tolerant in low-salinity environments (Xia et al., 2017a). In the outer bay, salinity was lower in summer and autumn compared to winter and spring. The lowest salinity and smallest proportion of type 3 coincided in October. RDA analysis exhibited a positive correlation between type 3 and salinity, indicating the importance of salinity in type 3 distribution. Although the relationship between type 3 and nutrients is still unclear, RDA analysis exhibited a negative correlation between type 3 and nutrients NO_3_^−^, NO_2_^−^, NH_4_^+^, and PO_4_^3−^. Nutrients in summer and autumn were generally higher than those in winter and spring, especially for PO_4_^3−^, phosphorus stress was obvious in winter and spring and relieved in October. The mechanisms of temperature, salinity, and nutrients influencing *Synechococcus* pigment types are still unclear, which is necessary to figure out the next step.

### *Synechococcus* pigment types comparison measured by different methods (FCM and *cpcBA* sequencing)

Flow cytometry has been widely used for the measurement of *Synechococcus* abundance since the 1980s (Olson et al., 1988; Liu et al., 2014). PC-only *Synechococcus* (type 1) and PE-rich *Synechococcus* (type 2 + type 3) can be separated using flow cytometer with dual lasers of 488 nm and 640 nm (Murrel and Lores, 2004; Liu et al., 2014). Type 1 could be recognized by red fluorescence induced by 640 nm laser because of the binding phycocyanobilin (PCB, Amax = 620 nm) (Six et al., 2007; Liu et al., 2014). PE-*Synechococcus* type 2 and type 3 could be separated by green fluorescence induced by 488 nm laser since type 3 showed higher green fluorescence than type 2 because of the binding phycourobilin (PUB, *A*_max_ = 495 nm) (Olson et al., 1988). In this study, *Synechococcus* type 1, type 2, and type 3 were clearly discriminated by dual lasers of a flow cytometer in Jiaozhou Bay (Figure 2). This was the first time three pigment types of *Synechococcus* were simultaneously distinguished. In the Hong Kong coastal waters and Pearl River Estuary, PC-only (type 1) and PE-rich (type 2 + type 3) *Synechococcus* were detected, whereas, in the Bohai Sea, Yellow Sea, and the East China Sea, only PE-Synechococcus was detected (Liu et al., 2014; Xia et al., 2017a, 2018; Wang et al., 2021, 2022a, 2022c).

In this study, both flow cytometric analysis and phylogenetic analysis of the *cpcBA* operons were applied to distinguish *Synechococcus* pigment types type 1, type 2, and type 3. 9 in 12 samples exhibited a similar distribution pattern, which showed 75% similarity of the two above methods, implying the reliability of *Synechococcus* pigment types analyzed by flow cytometry. The dissimilarity mainly comes from three samples in winter and spring in the inner bay (Feb-C1, May-C1, and May-A5) with low abundances (<10^3^ cells/mL) (Supplementary Figure S2). A low abundance of *Synechococcus* analyzed by flow cytometer may confuse in recognizing type 2 and type 3 by operators. Besides, a small proportion of dividing cells with replicated chromosomes measured by molecular method may be detected as single cells by flow cytometer (Tai and Palenik, 2009). This may cause a slight mismatch between the two above methods. To improve the accuracy of flow cytometric detection, it is necessary to enrich *Synechococcus* abundances collected in winter and spring by filtering before analysis and increasing the acquisition time during analysis. On the other hand, there is a need to use more sensitive and efficient molecular methods, such as qPCR for quantitative analysis of specific groups of *Synechococcus*, making the results more comparable and accurate (Wang et al., 2022c).

### Spatio-temporal variation of *Synechococcus* genetic diversity (*rpoC1*)

In the present study, 10 clades representing *Synechococcus* S5.1, S5.2, and S5.3 were identified (Figure 7A). S5.1 and S5.2 were abundant and widespread in the temperate Jiaozhou Bay. In comparison, 6–13 *Synechococcus* clades were identified in the temperate Chesapeake Bay (Chen et al., 2006), Gulf of Aqaba (Post et al., 2011), and Yellow Sea (Wang et al., 2021). In the subtropical Hong Kong waters, 17 clades were identified with abundant S5.1 and S5.2 in the coastal and estuarine waters (Xia et al., 2015). Our results were consistent with previous reports mentioned above.

Temporal and spatial variations in the distribution of the *Synechococcus* assemblages have been reported in various estuarine and coastal waters. *Synechococcus* population did not vary obviously and was dominated by a single clade, for instance in the Gulf of Aqaba, S5.1-II was predominant over a 3 years scale throughout the oceanic region (Fuller et al., 2005). At the Martha’s Vineyard Coastal Observatory (MVCO), S5.1-I dominated *Synechococcus* populations all over the year (Hunter-Cevera et al., 2016). However, in the subtropical Hong Kong waters, dominant lineages of *Synechococcus* were multiple and varied with seasons and locations. From spring to summer, S5.2 dominated in the estuarine water whereas S5.1-II and S5.1-VI co-dominated in coastal waters. In winter S5.1-II and S5.1-IX co-dominated in the estuarine and coastal waters (Xia et al., 2015). In Jiaozhou Bay, *Synechococcus* assemblages varied mainly with seasons. The composition of *Synechococcus* assemblages shifted from two dominant lineages in winter and spring to a high genetic diversity in summer and autumn. Three major lineages S5.1-I, S5.1-IX, and S5.2-CB5 were detected, with S5.1-I and S5.1-IX being dominant in winter and spring, and in autumn, respectively. S5.2-CB5 was abundant and widespread in most samples (Figure 7A). S5.1-I was dominant in winter and spring with temperatures ≤16°C. The importance of S5.1-I decreased with increasing temperature (Figure 7B). RDA analysis showed that S5.1-I was negatively correlated with temperature, indicating the importance of temperature in regulating S5.1-I distribution (Figure 8B). Similarly at MVCO, S5.1-I has been reported to be predominant over the entire year (Hunter-Cevera et al., 2016). S5.1-I is known as a cold-water *Synechococcus*, which is primarily found in cold and temperate, nutrient-rich coastal waters at higher latitudes. Higher tolerance to cold stress together with the ability to survive in low temperatures may be favorable for the dominance of S5.1-I in cold water (Hunter-Cevera et al., 2016; Doré et al., 2022).

S5.1-I usually co-occurred with S5.1-IV in the cold water. However, in Jiaozhou Bay, S5.1-IV was not detected. S5.1-I co-dominated with S5.2-CB5 in winter and spring. S5.2-CB5 was abundant and widespread in most samples, especially in summer in the inner bay, S5.2-CB5 was predominant with relative abundance >50% (Supplementary Table S6). S5.2-CB5 was always found in coastal and estuarine waters (Cai et al., 2010; Huang et al., 2012; Hunter-Cevera et al., 2016). Temperature, salinity, and nutrient concentrations might be important in regulating S5.2-CB5 distribution. In the Chesapeake Bay, S5.2-CB5 was merely detected in summer and dominated in the upper bay, which was characterized by a higher ammonium concentration and lower salinity (Chen et al., 2006; Cai et al., 2010). At the MVCO, S5.2-CB5 was isolated during later summer and fall, when the temperature was relatively high (17–20°C) and nitrate + nitrite concentration was relatively low (<0.5 μM) (Hunter-Cevera et al., 2016). Similarly in Jiaozhou Bay, the dominant *Synechococcus* lineage S5.2-CB5 was observed in summer with high temperature (28.9–29.5°C), low salinity (27.2–29.5), and low ammonium (0.36–1.57 μM) (Figure 7B). RDA analysis showed that S5.2-CB5 was negatively correlated with NO_3_^−^, NH_4_^+^ and PO_4_^3−^, implying the importance of nutrients in S5.2-CB5 regulation (Figure 8B). Representative of S5.2-CB5 showed a pigment type 1, which may largely contribute to the presence of type 1 in Jiaozhou Bay.

S5.1-IX exhibited an opposite trend compared to S5.1-I, being dominant in summer and autumn. Especially in autumn, S5.1-IX was predominant with relative abundance >35.7% (Supplementary Table S6). Unlike S5.1-I and S5.2-CB5, S5.1-IX was not a common *Synechococcus* clade, the knowledge about S5.1-IX was rather scarce. S5.1-IX was first discovered in the Gulf of Aqaba (Fuller et al., 2003). Nevertheless, it was considered to be rare in this field (Penno et al., 2006). In the China Sea, S5.1-IX was detected as a minor component in the East China Sea and the Yellow Sea (Choi and Noh, 2009; Chung et al., 2015; Wang et al., 2021). Even in the global ocean, Clade IX was reported to have low abundance (less than 5%) (Zwirglmaier et al., 2008). However, it thrived in the subtropical Hong Kong waters in October and December (Xia et al., 2015). Pyrosequencing analysis and growth experiment indicated that S5.1-IX (MW02) prefers to grow in low salinity waters and grows best at a salinity of 28 in Hong Kong waters (Xia et al., 2015). In the temperate Jiaozhou Bay, salinities ranged from 28.5 to 30.3 in autumn, which might be suitable for the growth of Clade IX. In autumn, phosphorus stress was largely relieved owing to higher nitrogen and phosphate concentrations. Sufficient nutrients may be another key factor for Clade IX growth in autumn. RDA analysis indicated that S5.1-IX was positively related to NO2-and temperature, and negatively related to salinity (Figure 8B). Compared to the environmental parameters, it seemed higher temperature, lower salinity, and sufficient nutrients could be the important factors for the dominance of S5.1-IX in autumn (Figure 7B). Since environmental conditions in the temperate Jiaozhou Bay are obviously different from those in the subtropical Hong Kong waters, S5.1-IX in Jiaozhou Bay might be a different subclade. It is necessary to isolate and sequence the clade S5.1-IX in Jiaozhou in the next step.

### The relationship of *Synechococcus* pigment types (FCM, *cpcBA*) and genetic diversity (*rpoC1*)

In our study, *Synechococcus* pigment types based on flow cytometry analysis and phylogenetic analysis of *cpcBA* operon showed similar distribution patterns in most samples. Liu et al. (2014) also showed phylogenetic study based on *cpcBA* agreed well with flow cytometric counts, which revealed the coexistence of PC-rich and PE-rich *Synechococcus* in the subtropical coastal waters.

Our phylogenetic tree based on *cpcBA* operon sequences, formed three well-separated clusters (type 1, type 2, and type 3) (Supplementary Figure S1). Type 1 corresponds to S5.1-VIII and S5.2; Type 2 corresponds to clades II, V, and VI of S5.1; Type 3 corresponds to clades I, II, III, IV, and WPC1 of S5.1. Based on the phylogeny of *rpoC1* gene, clades I, II, III, V, VI, VIII, IX, WPC1 of S5.1, S5.2, and S5.3 were detected (Figure 6). The phylogenic tree based on the two genes showed congruency. Comparing their responses to environmental factors, type 1 *Synechococcus* (S5.1-VIII, S5.2) based on *cpcBA* operon was positively correlated with Chl *a*, which was consistent with S5.1-VIII and S5.2 based on *rpoC1* gene (Figure 8). This fully indicates that *Synechococcus* type 1 in Jiaozhou Bay was mainly contributed by S5.1-VIII and S5.2, accompanied by high Chl *a* concentration, which is consistent with the spatio-temporal distribution of *Synechococcus* type 1 mentioned above (Figures 3, 5). However, the relationship between *Synechococcus* type 2 and type 3 and environmental factors, is inconsistent with the results based on *rpoC1* gene. The reason is that the single clade of *Synechococcus* assemblages may possess phycobilisomes of different types, i.e., S5.1-II corresponds to type 2 and type 3, consistent with observations on isolates (Haverkamp et al., 2008; Everroad and Wood, 2012; Xia et al., 2017b). *Synechococcus* clades have higher diversity compare to pigment types (Supplementary Table S5). The response of different *Synechococcus* clades to environmental factors will be more detailed than that of pigment types. The combination of more technical means provides more information for studying *Synechococcus* distribution.

## Conclusion

This present study investigated seasonal and spatial variations of *Synechococcus* in abundance, pigment types and genetic diversity in the temperate Jiaozhou Bay. *Synechococcus* abundance exhibited seasonal variations with highest value in summer and lowest value in winter, which was consistent with temperature variation. Three pigment types of *Synechococcus* type 1, type 2, and type 3 were discriminated simultaneously by dual lasers of flow cytometry for the first time. The phylogenetic analysis of *cpcBA* operon also revealed three pigment types of *Synechococcus* type 1, type 2, and type 3. By comparison, 75% similarity was shown between the two approaches, indicating the reliability of pigment types measured by flow cytometry. It is essential to eliminate the 25% mismatch between the two methods. We will attempt in various aspects, for instance, by increasing acquisition cells and time of flow cytometry and applying more sensitive and efficient molecular methods. Three pigment types of *Synechococcus* type 1, type 2 and type 3 were distinguished based on *cpcBA* operon, which displayed obvious variations spatially between the inner bay and the outer bay. Freshwater discharge and water turbidity played important roles in regulating *Synechococcus* pigment types. *Synechococcus* assemblages were phylogenetically diverse (12 different lineages) based on *rpoC1* gene and dominated by three core lineages S5.1-I, S5.1-IX, and S5.2-CB5 in different seasons, which were influenced by different environmental factors.

From this study, we clearly know about Synechococcus abundance, pigment types and genetic diversity on a spatio-temporal scale by different techniques in Jiaozhou Bay. The combination of more technical means provides more information for studying *Synechococcus* distribution. However, more questions are raised and need to be addressed. (1) Since S5.2-CB5 was widespread in all the seasons, it might be the best-fit genotype for Jiaohou Bay, which could be monitored as an indicator for long-term investigation. (2) The subclade of S5.1-IX in Jiaozhou Bay is still unclear. It is urgent to isolate, culture and sequence the S5.1-IX strains in Jiaozhou Bay and compare them with the subclade (NW02) in the subtropical Hong Kong strains.

## Data availability statement

The datasets presented in this study can be found in online repositories. The names of the repository/repositories and accession number(s) can be found at: https://www.ncbi.nlm.nih.gov/, BioProject PRJNA996355, https://www.ncbi.nlm.nih.gov/, BioProject PRJNA995918.

## Author contributions

SL: Data curation, Formal analysis, Methodology, Software, Visualization, Writing – original draft. YD: Conceptualization, Formal analysis, Methodology, Writing – original draft. XS: Conceptualization, Resources, Writing – review & editing. YZ: Conceptualization, Funding acquisition, Supervision, Writing – review & editing, Visualization. LZ: Conceptualization, Funding acquisition, Methodology, Supervision, Visualization, Writing – original draft, Writing – review & editing. WZ: Conceptualization, Supervision, Writing – review & editing. TX: Conceptualization, Supervision, Writing – review & editing.

## Footnotes

^1^ https://www.omicstudio.cn/tool/62
