# Supplementary material for: Seasonal and spatial variations of Synechococcus in abundance, pigment types, and genetic diversity in a temperate semi-enclosed bay
Source: Front Microbiol. 2024 Jan 11;14:1322548. doi: 10.3389/fmicb.2023.1322548 (PMC10808157; doi:10.3389/fmicb.2023.1322548)
Supplement: Supplementary file 1 [file Data_Sheet_1.docx]

Supplementary Material

# Supplemental material figures





Supplementary Figure 1. Phylogenetic analysis of representative *cpcBA* operon sequences of the 30 most abundant OTUs. Heatmap indicates the relative abundance of each OTU (Log transformed). The left bars indicate different pigment types. Bootstrap values larger than 50% were shown.


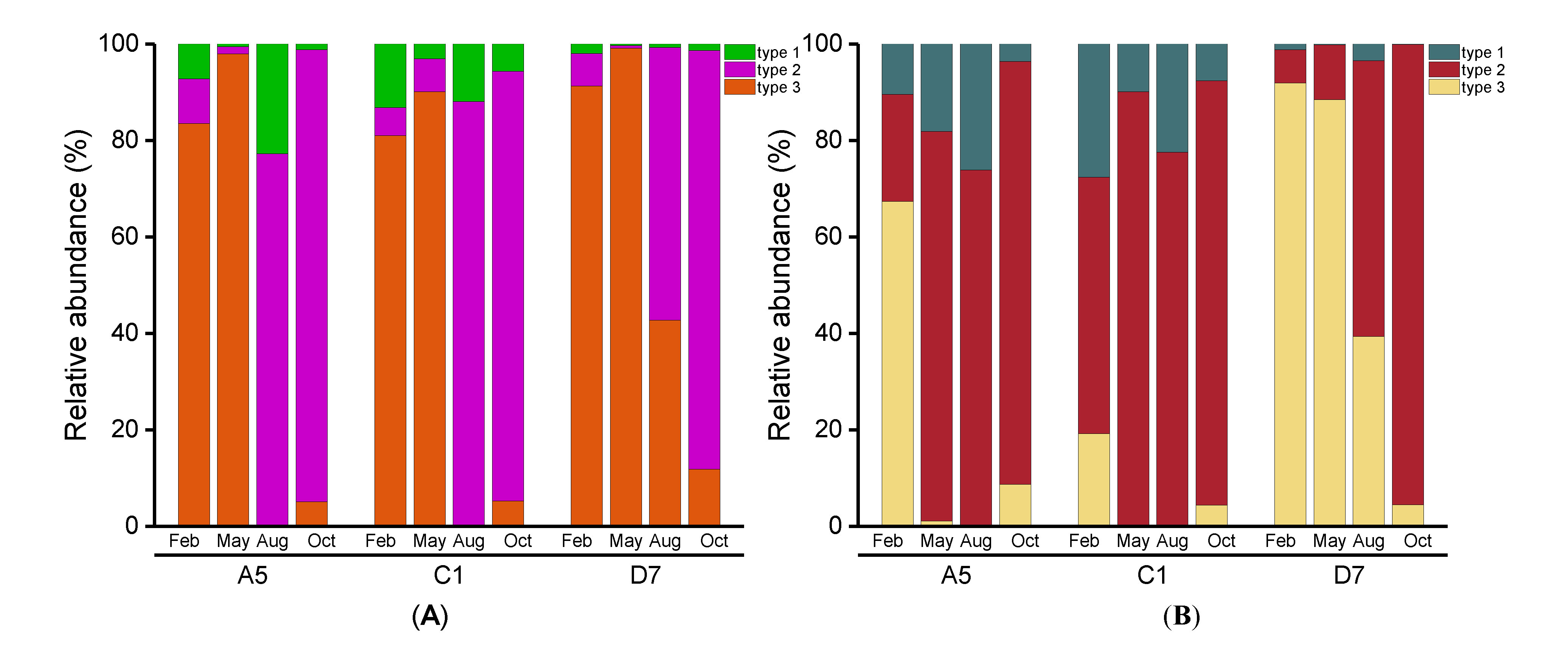


Supplementary Figure 2. The relative abundance of *Synechococcus* pigment types based on flow cytometric analysis (A) and *cpcBA* operon (B), respectively.

# Supplemental material tables

Supplementary Table 1. Reference sequences of *cpcBA* gene.

| Representative strain | Cluster | Clade | Pigment type | Accession number |
| --- | --- | --- | --- | --- |
| *Synechococcus* sp. CB0101 | S5.2 | CB4 | 1 | NZ_CP039373.1:1949345-1949833 |
| *Cyanobium* sp. NS01 | S5.2 |  | 1 | KF528824.1 |
| *Cyanobium* sp. PCC6307 | S5.2 |  | 1 | NC_019675.1:1021050-1021541 |
| *Cyanobium* sp. PCC7001 | S5.2 |  | 1 | NZ_DS990556.1:1499858-1500346 |
| *Synechococcus* sp. WH5701 | S5.2 |  | 1 | NZ_CH724160.1:1030769-1031260 |
| *Synechococcus* sp. RS9909 | S5.1 | VIII | 1 | KF528823.1 |
| *Synechococcus* sp. RS9917 | S5.1 | VIII | 1 | NZ_CH724158.1:604220-604708 |
| *Synechococcus* sp. WH8101 | S5.1 | VIII | 1 | KF528825.1 |
| *Synechococcus* sp. A15-44 | S5.1 | II | 2 | KF528820.1 |
| *Synechococcus* sp. BMK-MC-1 | S5.1 | V | 2 | KF528822.1 |
| *Synechococcus* sp. WH7805 | S5.1 | VI | 2 | NZ_CH724168.1:1142506-1142994 |
| *Synechococcus* sp. PROS-7-1 | S5.1 | VI | 2 | KF528821.1 |
| *Synechococcus* sp. ROS8604 | 5.1 | I | 3a | KF528803.1 |
| *Synechococcus* sp. SYN20 | 5.1 | I | 3a | MG014518.1 |
| *Synechococcus* sp. M16.1 | 5.1 | II | 3a | KF528812.1 |
| *Synechococcus* sp. RS9907 | 5.1 | II | 3a | KF528813.1 |
| *Synechococcus* sp. A15-24 | 5.1 | III | 3c | MG014524.1 |
| *Synechococcus* sp. A15-127 | 5.1 | WPC1 | 3c | KF528804.1 |
| *Synechococcus* sp. WH8102 | 5.1 | III | 3c | NC_005070.1:1921830-1922318 |
| *Synechococcus* sp*.* Minos11 | S5.3 |  | 3dB | KF528826.1 |
| *Synechococcus* sp. A15-62 | 5.1 | II | 3dB | KF528815.1 |
| *Synechococcus* sp. KORDI-52 | 5.1 | II | 3bB | NZ_CP006271.1:47777-48265 |
| *Synechococcus* sp. WH8103 | 5.1 | III | 3bB | KF528809.1 |
| *Synechococcus* sp. BIOS-U3-1 | 5.1 | CRD1 | 3dA | KF528806.1 |
| *Synechococcus* sp. CC9902 | 5.1 | IV | 3dA | NC_007513.1:1826501-1826989 |

Supplementary Table 2. Reference sequences of *rpoC1* gene.

| Representative strain | Cluster | Clade | Accession number |
| --- | --- | --- | --- |
| *Synechococcus* sp.st235 | S5.1 | I | AF448104.1 |
| *Synechococcus* sp. WH8016 | S5.1 | I | GU990533.1 |
| *Synechococcus* sp. ROS8604 | S5.1 | I | CP047946.1 |
| *Synechococcus* sp.CC9311 | S5.1 | I | AF013607.1 |
| *Synechococcus* sp.CC9617 | S5.1 | I | AF154562.1 |
| *Synechococcus* sp.58E8 | S5.1 | II | AF448101.1 |
| *Synechococcus* sp.RS9911 | S5.1 | II | AJ621009.1 |
| *Synechococcus* sp. WH6501 | S5.1 | II | AF448106.1 |
| *Synechococcus* sp.CC9301 | S5.1 | III | AF153332.1 |
| *Synechococcus* sp.C129 | S5.1 | III | AF153339.1 |
| *Synechococcus* sp.CC9703 | S5.1 | III | AF153338.1 |
| *Synechococcus* sp. CB-1 | S5.1 | IV | GU990581.1 |
| *Synechococcus* sp. UW01 | S5.1 | V | AJ621020.1 |
| *Synechococcus* sp. WH7803 | S5.1 | V | L34061.1 |
| *Synechococcus* sp. WH8018 | S5.1 | VI | AJ621015.1 |
| *Synechococcus sp*. WH7805 | S5.1 | VI | L34062.1 |
| *Synechococcus* sp.1002 | S5.1 | VI | AF448082.1 |
| *Synechococcus* sp. UW92 | S5.1 | VII | JQ421053.1 |
| *Synechococcus* sp.RS9920 | S5.1 | VII | AJ621012.1 |
| *Synechococcus* sp.RS9906 | S5.1 | VIII | JQ421058.1 |
| *Synechococcus* sp.RS9914 | S5.1 | VIII | AJ621011.1 |
| *Synechococcus* sp.59 | S5.1 | IX | AF448100.1 |
| *Synechococcus* sp.RS9901 | S5.1 | IX | AJ621003.1 |
| *Synechococcus* sp. UW106 | S5.1 | XV | JQ421054.1 |
| *Synechococcus* sp. KORDI-49 | S5.1 | WPC1 | CP006270.1 |
| *Synechococcus* sp. WH8007 | S5.2 | CB5 | AF448107.1 |
| *Synechococcus* sp. Minos01 | S5.3 |  | AJ621022.1 |
| *Synechococcus* sp. Minos11 | S5.3 |  | AJ621021.1 |
| *Synechococcus* sp. PCC6307 | *Cyanobium* |  | U52342.1 |
| *Synechococcus* sp. PCC9005 | *Cyanobium* |  | AF245160.1 |
| *Synechococcus* sp.PS841 | *Cyanobium* |  | AF448092.1 |
| *Synechococcus* sp.PS719 | FS-I |  | AF245141.1 |
| *Synechococcus* sp.PS675 | FS-II |  | AF245148.1 |
| *Synechococcus* sp.PS676 | FS-II |  | AF245135.1 |

Supplementary Table 3. Spearman correlation analysis of *Synechococcus* abundance (i.e., type 1, type 2, type3, and Total ABU) and environmental variables (i.e., temperature, salinity, Chl *a*, PO_4_^3-^, NO_2_^-^, NO_3_^-^, NH_4_^+^). Abbreviations used in the table: ABU = abundance, T = Temperature, S = Salinity. ** indicate significant differences (p < 0.01).

|  | type 1 ABU | type 2 ABU | type 3 ABU | Total ABU |
| --- | --- | --- | --- | --- |
| T | 0.84 ^**^ | 0.94 ^**^ | 0.41 | 0.93 ^**^ |
| S | -0.86 ^**^ | -0.80 ^**^ | -0.03 | -0.68 ^**^ |
| Chl *a* | 0.30 | 0.23 | -0.51 | 0.10 |
| PO_4_^3-^ | 0.26 | 0.31 | 0.08 | 0.16 |
| NO_2_^-^ | 0.54 | 0.50 | -0.25 | 0.38 |
| NO_3_^-^ | 0.40 | 0.32 | -0.14 | 0.22 |
| NH_4_^+^ | 0.11 | -0.04 | -0.27 | 0.13 |

Supplementary Table 4. The result of Kruskal-Wallis test and pair-wise tests with two factors (season and station) to compare different *Synechococcus* abundance (i.e., type 1, type 2, type 3, and Total ABU) in Jiaozhou Bay. Abbreviations used in the table: ABU = abundance. * indicate significant differences (p < 0.05); ** indicate significant differences (p < 0.01).

| *Synechococcus* ABU types | Factors | p |
| --- | --- | --- |
| Total SYN | season | 0.016^*^ |
|  | station | 0.981 |
| type 1 SYN | season | 0.03^*^ |
|  | station | 0.469 |
| type 2 SYN | season | 0.019^*^ |
|  | station | 0.794 |
| type 3 SYN | season | 0.086 |
|  | station | 0.309 |

Pair-wise tests for the four seasons

| *Synechococcus* ABU type |  | p |
| --- | --- | --- |
| Total SYN | winter & spring | 0.308 |
|  | winter & autumn | 0.042^*^ |
|  | winter & summer | 0.002^**^ |
|  | spring & autumn | 0.308 |
|  | spring & summer | 0.042^*^ |
|  | autumn & summer | 0.308 |
| type 1 SYN | winter & spring | 0.910 |
|  | winter & autumn | 0.089 |
|  | winter & summer | 0.013^*^ |
|  | spring & autumn | 0.112 |
|  | spring & summer | 0.017^*^ |
|  | autumn & summer | 0.427 |
| type 2 SYN | winter & spring | 0.428 |
|  | winter & autumn | 0.054 |
|  | winter & summer | 0.003^**^ |
|  | spring & autumn | 0.258 |
|  | spring & summer | 0.031^*^ |
|  | autumn & summer | 0.308 |

Supplementary Table 5. Sequencing information and alpha diversity indices of *cpcBA* and *rpoC1* gene.

| Samples | *cpcBA* | | | *rpoC1* | | |
| --- | --- | --- | --- | --- | --- | --- |
|  | Number of sequences | Coverage | Shannon | Number of sequences | Coverage | Shannon |
| Feb-A5 | 9919 | 0.99 | 2.61 | 9365 | 0.99 | 4.06 |
| May-A5 | 12370 | 0.99 | 1.82 | 7213 | 0.99 | 4.35 |
| Aug-A5 | 11271 | 0.99 | 2.26 | 2497 | 0.99 | 3.97 |
| Oct-A5 | 7847 | 0.99 | 3.11 | 2551 | 0.99 | 4.48 |
| Feb-C1 | 7171 | 0.99 | 3.98 | 7822 | 0.99 | 4.45 |
| May-C1 | 10220 | 0.99 | 1.59 | 6039 | 0.99 | 4.19 |
| Aug-C1 | 9680 | 0.99 | 2.20 | 2560 | 0.99 | 4.24 |
| Oct-C1 | 9376 | 0.99 | 3.19 | 2670 | 0.99 | 4.87 |
| Feb-D7 | 16207 | 0.99 | 1.21 | 11595 | 0.99 | 3.79 |
| May-D7 | 12397 | 0.99 | 2.30 | 7209 | 0.99 | 4.01 |
| Aug-D7 | 11975 | 0.99 | 2.63 | 3717 | 0.99 | 4.02 |
| Oct-D7 | 6876 | 0.99 | 2.41 | 793 | 0.97 | 3.76 |

Supplementary Table 6. Relative abundance of each lineage based on *rpoC*1 sequencing in 12 samples.

| Lineage | Sample | | | | | | | | | | | | |
| --- | --- | --- | --- | --- | --- | --- | --- | --- | --- | --- | --- | --- | --- |
|  | Group1 | | | | | | Group2 | Group3 | | Group4 | | |  |
|  | 2-C1 | 2-A5 | 2-D7 | 5-D7 | 5-C1 | 5-A5 | 8-D7 | 8-C1 | 8-A5 | 10-C1 | 10-A5 | 10-D7 | |
| S5.1-I | 71.94 | 81.21 | 87.88 | 81.78 | 41.62 | 66.30 | 42.12 | 1.82 | 2.12 | 1.74 | 1.41 | 1.32 | |
| S5.1-IX | 1.02 | 1.01 | 0.33 | 0.41 | 1.26 | 0.52 | 13.33 | 29.86 | 26.07 | 35.74 | 42.64 | 44.97 | |
| S5.1-II | 0.04 | 0.00 | 0.01 | 0.02 | 0.00 | 0.00 | 0.46 | 0.51 | 0.17 | 5.43 | 6.19 | 11.11 | |
| S5.1-III | 0.25 | 0.02 | 0.00 | 0.00 | 0.00 | 0.10 | 4.92 | 0.51 | 0.04 | 1.79 | 0.47 | 0.79 | |
| S5.1-V | 0.04 | 0.00 | 0.00 | 0.00 | 0.00 | 0.02 | 0.03 | 1.07 | 0.09 | 0.11 | 0.00 | 0.00 | |
| S5.1-VI | 1.68 | 0.03 | 0.04 | 0.00 | 0.06 | 0.00 | 9.40 | 1.03 | 0.17 | 0.90 | 0.67 | 0.00 | |
| S5.1-VIII | 0.83 | 0.00 | 0.00 | 0.02 | 0.00 | 0.00 | 0.03 | 1.15 | 3.12 | 0.90 | 0.13 | 0.13 | |
| S5.1-WPC1 | 0.00 | 0.00 | 0.00 | 0.00 | 0.00 | 0.00 | 0.00 | 0.04 | 0.00 | 1.40 | 1.75 | 2.51 | |
| S5.1 sum (all clade) | 75.79 | 82.26 | 88.26 | 82.23 | 42.94 | 66.94 | 70.28 | 36.00 | 31.79 | 48.01 | 53.26 | 60.85 | |
| S5.2-CB5 | 17.67 | 14.65 | 11.16 | 17.68 | 51.93 | 32.28 | 28.65 | 49.98 | 61.41 | 26.61 | 24.95 | 30.42 | |
| S5.3 | 0.00 | 0.00 | 0.00 | 0.02 | 0.00 | 0.00 | 0.03 | 0.67 | 0.00 | 6.67 | 18.09 | 7.80 | |
| *Cyanobium* | 0.60 | 1.23 | 0.09 | 0.00 | 0.00 | 0.00 | 0.00 | 0.12 | 0.09 | 0.17 | 0.00 | 0.00 | |
| FS | 2.49 | 0.69 | 0.17 | 0.02 | 4.51 | 0.64 | 0.00 | 1.03 | 3.46 | 2.58 | 0.20 | 0.40 | |
| Unclassied | 3.45 | 1.18 | 0.32 | 0.06 | 0.62 | 0.15 | 1.04 | 12.20 | 3.25 | 15.97 | 3.50 | 0.53 | |
